# Transfer learning with class activation maps in compositions driving plaque classification in carotid ultrasound

**DOI:** 10.3389/fdgth.2025.1484231

**Published:** 2025-07-09

**Authors:** Georgia D. Liapi, Christos P. Loizou, Maura Griffin, Constantinos S. Pattichis, Andrew Nicolaides, Efthyvoulos Kyriacou

**Affiliations:** ^1^ e-Health Laboratory, Department of Electrical Engineering, Computer Engineering and Informatics, Cyprus University of Technology, Limassol, Cyprus; ^2^ Vascular Screening and Diagnostic Centre, Nicosia, Cyprus; ^3^ e-Health Laboratory, Department of Computer Science, University of Cyprus, Nicosia, Cyprus; ^4^ HealthXR Research Group, CYENS Centre of Excellence, Nicosia, Cyprus

**Keywords:** transfer learning, carotid ultrasound, plaque, attention maps, compositions

## Abstract

**Introduction:**

Carotid B-mode ultrasound (U/S) imaging provides more than the degree of stenosis in stroke risk assessment. Plaque morphology and texture have been extensively investigated in U/S images, revealing plaque components, such as juxtaluminal black areas close to lumen (JBAs), whose size is linearly related to the risk of stroke. Convolutional neural networks (CNNs) have joined the battle for the identification of high-risk plaques, although the ways they perceive asymptomatic (ASY) and symptomatic (SY) plaque features need further investigation. In this study, the objective was to assess whether class activations maps (CAMs) can reveal which U/S grayscale-(GS)-based plaque compositions (lipid cores, fibrous content, collagen, and/or calcified areas) *influence* the model's understanding of the ASY and SY cases.

**Methods:**

We used Xception via transfer learning, as a base for *feature extraction* (all layers frozen), whose output we fed into a new dense layer, followed by a new classification layer, which we trained with standardized B-mode U/S longitudinal plaque images. From a total of 236 images (118 ASY and 118 SY), we used 168 in training (84 ASY and 84 SY), 22 in internal validation (11 ASY and 11 SY), and 46 in testing (23 ASY and 23 SY).

**Results:**

In testing, the model reached an accuracy, sensitivity, specificity, and area under the curve at 80.4%, 82.6%, 78.3%, and 0.80, respectively. Precision and the F1 score were found at 81.8% and 80.0%, and 79.2% and 80.9%, for the ASY and SY cases, respectively. We used faster-Score-CAM to produce a *heatmap* for each tested image, quantifying each plaque composition area overlapping with the heatmap to find compositions areas related to ASY and SY cases. Dark areas (GS ≤ 25) or JBAs (whose presence was verified priorly, by an experienced vascular surgeon) were found *influential* for the understanding of both the ASY and the SY plaques. Calcified areas, fibrous content, and lipid cores, *together*, were more related to ASY plaques.

**Conclusions:**

These findings indicate the need for further investigation on how the GS ≤ 25 plaque areas affect the learning process of the CNN models, and they will be further validated.

## 1 Introduction

The global incidence and burden of ischemic stroke saw a substantial increase from 1990 to 2021 (87.69% and 101.8% increase, respectively) (1). Implementation of effective primary prevention pathways, after timely identification of individuals in high risk, for better disease outcomes and patient management, is needed. Although in (2), a thorough statistical analyses on, among others, stroke and cardiovascular risk factors has been provided, summarizing also habits contributing to cardiovascular health, the early identification and effective management of the asymptomatic (ASY) carotid stenosis degree (SD; when ≥70%) seem to remain an essential factor (3). According to Kamtchum-Tatuene et al. (3), plaques at high risk are common in ASY patients, while in another study, stroke risk was found approximately at 15% of patients with severe ASY stenosis (after 5 years of continuous medical therapy) (4), pointing to the reconsideration of surgical intervention.

B-mode ultrasound (U/S) imaging, a reliable imaging modality to monitor arterial wall thickness with good resolution, is widely preferred, mainly because of its cost-effectiveness and non-invasive nature (5). B-mode U/S enables more than the SD estimation, as proved by a series of studies relying on the Asymptomatic Carotid Stenosis and Risk of Stroke cohort data (6). Those studies exploited B-mode U/S image-based textural and morphological features of plaques, concluding to strong associations between echodensity configurations and ASY and symptomatic (SY) internal carotid artery (ICA) plaques, and plaque types (6–9). Another study also assessed B-mode U/S characteristics of *unstable* carotid plaques (10), where echolucent plaques were more present in patients with Amaurosis Fugax, Transient Ischaemic Attack, and stroke (86%) than in patients with ASY (47%, *p*-value < 0.001). In (11), a plethora of U/S plaque features were found, which change significantly throughout the cardiac cycle, between individuals with ASY and SY.

Researchers in (12) concluded that a high SD is related to more heterogeneous plaques, which were also found more associated with symptoms compared with homogeneous plaques, with any SD. However, later, researchers in (13) showed that *unstable* plaques appear as more echolucent and homogeneous (independently of the SD), based on associations between a combination of texture and echogenicity U/S plaque features and histologic plaque instability.

With the advent of artificial intelligence (AI), numerous deep learning (DL) models have been proposed and evaluated, with ImageNet (14), in binary and multiclass image classification, achieving remarkable classification accuracies. Some central examples were VGG (15), the ResNet-152 (16), and Xception (17). Multiple studies followed, training and evaluating DL models in medical image classification, pathological lesion detection, and segmentation tasks (18), showing promising performances, in many imaging modalities (19).

## 2 Related work

Transfer learning, a way to benefit from pretrained DL models, has also become popular in the automation of medical image classification tasks (20, 21). However, when used for a first time, with a medical image dataset, it requires meticulous fine-tuning of the pretrained model (22), as the efficacy of fine-tuning varies due to its intense dependence on model architecture and on the medical imaging modality. Although in medical image analysis, transfer learning has gained a notable ground, it comes with certain research gaps, such as the need for a better benchmarking or the need to comprehensively investigate what convolutional neural network (CNN) models understand, preferably through class activations visualizations (23). DL and transfer learning pathways, for the identification of ASY and SY plaques in carotid U/S images, supported by different ways of visualizations to show image areas that contribute to model's understanding, have been investigated in a limited amount of studies (Table 1). The idea of including, among others, class activation maps (CAMs) in CNN-based carotid U/S plaque classification workflows, has begun flourishing.

**Table 1:** Summary of previous research on deep and transfer learning-based carotid plaque classification in ultrasound images, combined with explainable class activation maps and other visualizations on the predictions, to investigate plaque compositions that mostly contribute to symptomatic, asymptomatic, low-risk, or high-risk plaques.

Year	Study	*N* subjects *N* images ASY/SY	Image preprocessing		Best CNN DL/TL	XAI method	Results			
			RN/INN	Input size			SE	SP	Mean CA (± STD)	Targeted composition or ASY/SY
2017	Lekadir et al. (24)	56 90,000^†^ NG/NG	Fixed/*	15 × 15	Custom CNN ✓/–	GT vs. Pred areas (mm^2^)	0.83 ± 0.12 0.70 ± 0.16 0.76 ± 0.15	0.90 ± 0.13 0.80 ± 0.14 0.89 ± 0.12	0.75 ± 0.16	Lipid core Fibrous cap calcified area
2021	Ganitidis et al. (25)	74 NG/NG 58/16	NG/NG	NG	Custom CNN ✓/–	Local surrogate models	0.75 ± 0.18 (4-f mean)	0.70 ± 0.10 (4-f mean)	0.73 ± 0.06 (balanced)	ASY vs. SY**
2021	Sanagala et al. (27)	346 400 150/196	Fixed/✓	224 × 224 256 × 256	MobileNet^‡^ Custom CNN ✓/✓	GradCAM on VGG16	NG	NG	0.962, 0.956	ASY vs. SY**
2024	Singh et al. (29)	190 223 0/190	Fixed/–	224 × 224	GoogLeNet ✓/-	GradCAM on GoogLeNet	0.986	0.976	0.982	Low vs. high risk
2024	He et al. (30)	1,339 3,860 –/– 674 1,564 –/–	Fixed/*	256 × 256	ResNet-50-based Custom CNN ✓/✓	GradCAM on ResNet-50-based Custom CNN	0.932^§^ 0.953^§§^ 0.816^§^ 0.685^§§^	0.992^§^ 0.822^§§^ 0.873^§^ 0.895^§§^	0.956^§^ 0.864^§§^ 0.837^§^ 0.746^§§^	Plaque Present vs. Absent Stable vs. Unstable
2024	Liapi et al. This study	232 236 116/116	✓/✓	512 × 224	Xception –/✓	f-ScoreCAM on Xception	0.826	0.783	0.804 (weighted average)	ASY vs. SY**

ASY, asymptomatic; CAM, class activation map; CNN, convolutional neural network; DL, deep learning; INN, intensity normalization; *N*, number; RN, resolution normalization; SE, sensitivity; SP, specificity; SY, symptomatic; TL, transfer learning; XAI, explainable artificial intelligence.

✓ The image intensity normalization method adhered to that in reference (52).

* The image intensity normalization method was different from that in reference (52).

** Classification of plaques as symptomatic or asymptomatic, with visualized insights on compositional components on the classified image.

^‡^ Multiple models were evaluated, pretrained on ImageNet; MobileNet returned their best area under the Receiver operating characteristic curve.

^§^ Internal validation dataset.

^§§^ External validation dataset.

Primarily, researchers in (24) had developed a small custom CNN model of four convolutional (CONV) layers, which they trained and evaluated for B-mode U/S carotid plaque tissue identification, taking into account the area around each pixel, as contained in image patches (large number of 15 × 15 pixel size patches), each hosting plaque tissue such as lipid cores, fibrous tissue, and/or calcified tissue. They visualized the predicted plaque tissues, calculating a pixel-wise accuracy, comparing them with ground truth (GT) composition areas. Later, researchers in (25) employed a slightly larger DL model (six CONV layers), which they trained with carotid U/S plaque images to differentiate between ASY and SY cases, and tested it, using local surrogate models (26) (local approximation of predictions) to interpret the model's decision. There, heatmaps showed areas that highly impacted a correctly classified ASY plaque and a misclassified SY plaque, reflecting the DL model's difficulty to confidently associate high-risk components, such as juxtaluminal black areas close to lumen (JBAs) (8), with the SY class.

Meanwhile, in (27), ten ImageNet-pretrained models were used, by replacing the initial top classification layers with new dense and classification layers, and trained and evaluated against a custom DL model (11 CONV layers), to classify ASY and SY plaques in carotid U/S images. There, MobileNet with transfer learning performed very well. Also, gradient-weighted class activation mapping (GradCAM) (28) gave heatmap visualizations in the tested images (when VGG16 was used), to explain the model's predictions. GradCAM pointed to echolucent areas for the SY class vs. more hyperechoic areas in the ASY plaques (only qualitatively assessed). Later in (29), researchers trained and evaluated five DL models with *high*- and *low-risk* plaques in carotid U/S images and used a support vector machine to classify a series of U/S plaque image features they extracted. There, GoogLeNet yielded the best results. They also used GradCAM on GoogLeNet to depict the most important plaque regions contributing to the model's understanding of high-risk plaques, without further quantification.

Finally, in (30), a DL model, with ResNet-50 as a backbone, was developed for carotid plaque *detection* and *stability* assessment, in two rounds of training with U/S images. There, researchers employed two single-input ResNet-50 architectures (image input in the former is a version where only edges are preserved, while input in the latter model is the original U/S image), whose outputs were fused (by bilinear combination) and fed into a dense layer to decide on plaque *presence* or *absence*. Then, a dual-input version of this model, sitting at the top of the plaque detection network, was trained to classify plaques into *stable* or *unstable*. There, researchers also used GradCAM to visualize areas that heavily contributed to the understanding of stable and unstable plaques, although in a qualitative manner.

Overall, visualizations of the above-mentioned areas, using CAMs, have constituted a more *explainable* pathway for humans to know what CNN models perceive as ASY or SY plaque-associated features, in B-mode U/S images. However, apart from (24, 25), the other above-mentioned studies provided qualitative visualizations. In (24, 25), there was further representation, assessment, and discussion on plaque compositions (lipid cores, fibrous content, calcifications, or JBAs), as detected by the CNNs. Importantly, in the past, the size of the JBA, without a visible echogenic cap, had been found linearly associated with the risk of stroke (8) (SD between 50% and 99%; treated medically), *upon image intensity normalization* (31), while other studies showed that JBAs > 6 mm^2^ possibly signify *vulnerable* plaques (32), as well as that a large JBA is associated with a higher ulceration score (33).

As included in the clinical practice guidelines, provided by the European Society for Vascular Surgery (ESVS) in 2023 (34), since the ESVS 2017 Guidelines, JBAs (grayscale value; GS ≤ 25) are considered among the recommended clinical imaging criteria to assess the risk of stroke. In addition, discrete white areas (6), representing calcified regions surrounded by hypoechoic regions, in plaques without acoustic shadow, are also associated with an increased risk of stroke (*p*-value < 0.001) (35). Presumably, the automatic and reliable identification and quantification of these critical plaque areas in U/S images could possibly allow an effective and timely stroke risk stratification, especially in ASY cases. As high-risk plaque areas might be present in patients with either ASY or SY, automatic classification into ASY and SY plaques might not be an adequate approach to depict the risk.

In this study, the aim was to investigate whether a CNN model (based on transfer learning) could identify plaque compositions as more ASY- or SY-specific. We performed carotid plaque classification, into ASY or SY, in B-mode U/S longitudinal images, utilizing a well-known pretrained CNN model, as *feature extractor*. From the model's last convolutional layer, we produced CAM heatmaps, and by keeping them as *uniform* maps, we quantified how *a*. the proportion of each plaque composition area covered by the map (with respect to each plaque composition), and *b*. the proportion of each plaque composition area in the map (with respect to the map), differ between the ASY and SY cases. Plaque compositions pertained to lipid cores, fibrous content, collagen and/or calcifications, given as GTs, in the form of six color contours. When CAMs are used in medical imaging tasks, they cannot always successfully point to truly affected lesions, due to high complexity. Here, the CAM maps served as interpretative means to detect the plaque composition areas involved, influencing the model's understanding, with a focus on quantifying their related area, rather than their overall precise localization.

## 3 Materials and methods

### 3.1 Carotid B-mode ultrasound image dataset

A total of 232 patients were included (116 ASY and 116 SY patients), from which 236 carotid B-mode U/S longitudinal images were available (for two SY and two ASY patients, recordings from the right and the left ICAs were available). Data information is given in Table 2. The majority of images corresponded to ICAs. The 236 carotid U/S videos were captured in three medical centers, in Cyprus, in the United Kingdom (UK), and in Greece. Overall, there was SD ≥ 50% [based on the European Carotid Surgery Trial protocol (ECST) for the cases from Cyprus and Greece or based on the North American Symptomatic Carotid Endarterectomy Trial protocol (NASCET) for the cases from UK]. An experienced vascular surgeon manually annotated the plaque in each U/S image, using a dedicated in-house software, providing us with GT plaques regions (36).

**Table 2:** Summary of the different carotid ultrasound image sources, for the data used in this study, along with the available patient demographics and the ultrasound machines.

Country	*N*_Patients_	*N*_ASY_	*N*_SY_	*N*_Females_	*N*_Males_	Age mean ± std	*N*_Images_	Ultrasound machine	Initial image resolution mean ± std (px/mm)
United Kingdom	186	103	83	69	117	73 ± 10	188	Philips iU22	14.29 ± 3.07
Cyprus	7	4	3	0	7	69 ± 9	7	Philips iE33 or Philips Affinity 70G	17.36 ± 2.81
Greece	26	9	17	11	15	73 ± 8	28	Philips iU22	14.12 ± 1.66
Greece	13	0	13	NG	NG	NG	13	ATL HDI-5000	17.00
Total	232	116	116	–	–	–	236^a^	–	–

ASY, asymptomatic; SY, symptomatic.

^a^ For two symptomatic and two asymptomatic patients, there were ultrasound recordings from both the right and the left internal carotid artery.

### 3.2 Carotid plaque composition ground truth areas

We produced 236 plaque image counterparts, based on *six color contours* (six ground truth colors*)*, to visualize each plaque composition. Each plaque composition was colored according to a prespecified GS intensity range, with a method introduced by Kyriacou and Nicolaides (36). These images served as GT carotid plaque composition areas, where JBAs (or lipid cores) are represented by *black* (GS ≤ 25), lipid cores with some amount of collagen (histologically it is fibro-fatty tissue) are shown in *blue* (25 < GS ≤ 50) and *green* (50 < GS ≤ 75), and calcified areas are depicted in *red* (GS > 125). We provide intermediate areas in *orange* (100 < GS ≤ 125) and *yellow* (75 < GS ≤ 100) for visualization purposes.

### 3.3 Data preparation and preprocessing

All U/S images were first resolution-normalized to 20 pixels/mm, and then intensity was normalized using linear scaling with two reference points (blood and adventitia), such that the grayscale median of blood was 0–5 and that of the adventitia was 185–190, according to the methods in (31, 37). Next, all images were cropped to closely keep the plaque (in image center) in its bounding box dimensions, including a limited surrounding U/S background. Finally, we resized all images to a uniform size, 512 × 224 pixels, before model training. This input dimensions were decided as the resolution-normalized *x*-axes of the plaques, in our dataset, were 277 ± 97 pixels long (max *x*-axis at 512), and the *y*-axes were at 96 ± 32 pixels (max *y*-axis at 201). For all computational processes and DL workflows, we worked in Python 3.8 (38).

### 3.4 Transfer learning carotid plaque classification with analyzed class attribution maps

**Figure 1:**
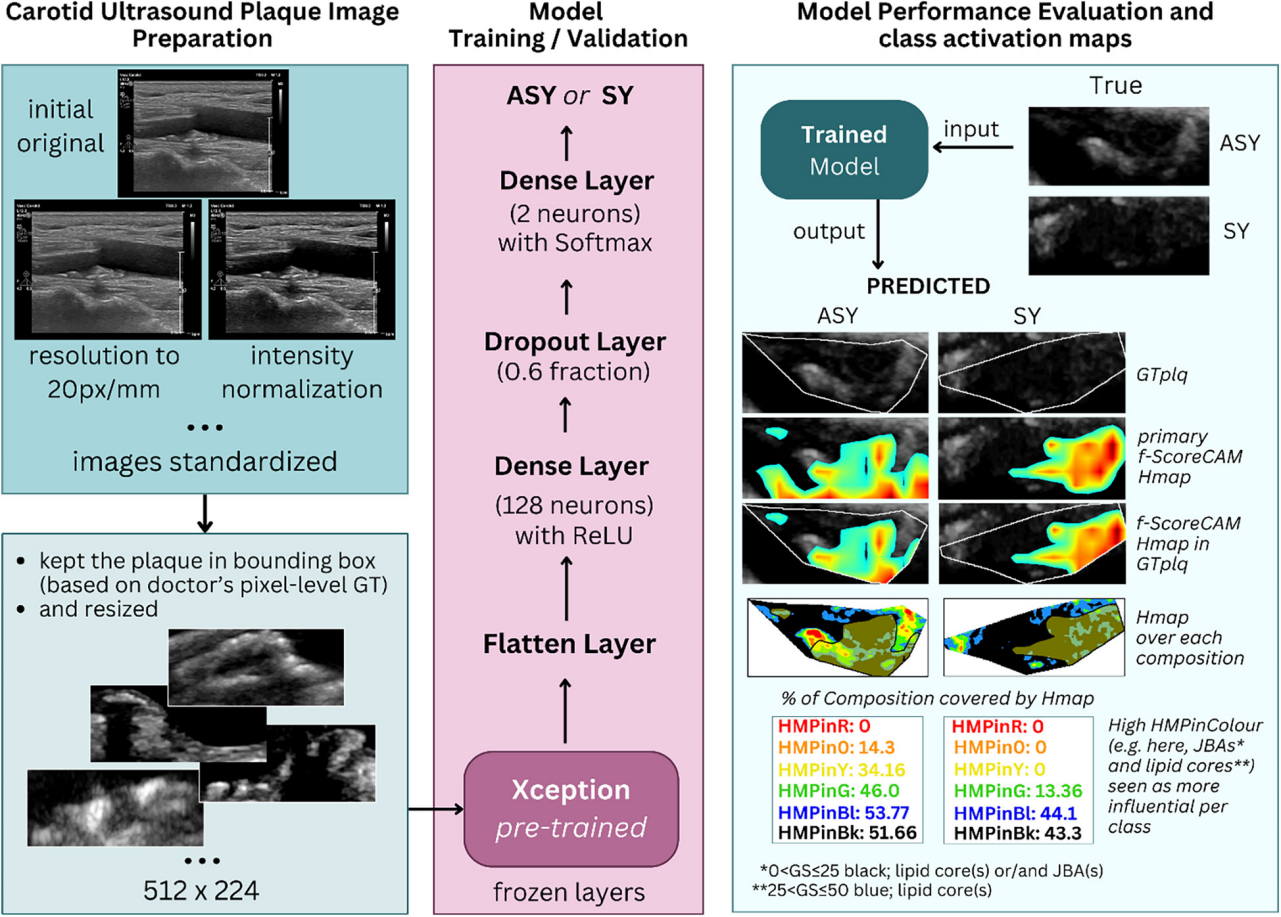
The process of transfer learning-based carotid plaque classification in B-mode longitudinal U/S images, as performed in this study, and plaque compositions involved in class understanding, revealed via f-Score-CAM. From left-to-right-to-left, we show how we used the pretrained Xception as feature extractor. After removal of the primary classification layer, we froze the backbone, and at the top of it, we added a new set of dense layers; the final was a classification layer, before which a dropout layer was set. We tested the model on kept-out images, we extracted the f-Score-CAM-based heatmap, we limited it to the plaque area bounding box and measured what proportion of the GTplq compositions was covered by the heatmap. ASY, asymptomatic; GS, grayscale; GT, ground truth; GTplq, ground truth plaque region; HMP, Heatmap; JBAs, juxtaluminal black areas close to lumen; SY, Symptomatic.

We used transfer learning with B-mode U/S plaque images, using the pretrained (on ImageNet; (39)) Xception (17) model, as a *feature extractor*, to classify carotid plaques into ASY or SY. We removed the primary classification layer of Xception (keeping all layers frozen), we flattened its output and added a new dense layer with 128 neurons (attempting not to introduce a larger number, to prevent overfitting), for training with our images, followed by a dropout layer and a new classification layer. We tested the model on our kept-out images and generated saliency maps and CAM-based heatmaps, employing SmoothGrad (40) and the faster-Score-CAM (f-Score-CAM) (41) (Section 3.5), respectively, to discover which plaque compositions the model associates with the ASY and SY classes. In Figure 1, we provide a holistic flow diagram of the transfer learning-assisted carotid plaque classification and the possible contribution of plaque compositions to this process, as conducted in the present study.

#### 3.4.1 Transfer learning model

We relied on Xception (17), which consists of 36 CONV layers, as it preserves a relatively good trade-off between top-1 accuracies and computational complexity (concerning model size, number of parameters, inference time and memory consumption, all together), based on the study of Bianco et al. (42). Here, we intended to evaluate a CNN model of a moderate size (but efficient), as the image dataset used here is not very large, and if used with a very large model, this would likely introduce overfitting. Xception can be understood as a linear stack of “depthwise separable convolutions” (DSCONVs), hosted within 14 modules. It consists of an *entry*, a *middle,* and an *exit* flow part. In the *entry* flow (two CONV layers and three residual blocks, with DSCONVs), complex patterns are read by the model, which gradually increases the depth to capture abstract features. In the *middle* flow, there are eight identical residual blocks, performing a repetitive refinement to understand the data, which led to the *exit* flow (residual blocks, average pooling and a fully-connected layer; FC), for classification. In the CONV blocks, there are also batch normalization layers, ReLU activations, and skip connections. Overall, Xception balances efficiency and better performance, compared with other previously introduced models.

#### 3.4.2 Model training and hyperparameter settings

Xception was used via Keras (43) [TensorFlow as backend (44)], as *feature extractor*. We created a model, with the pretrained Xception as the base (all layers frozen), to use its output to further train new dense layers, with *supervised* training. The new model was further comprised of a *Flatten* layer, a new dense layer (128 neurons; ReLU as activation function), a dropout layer (0.6 factor) to help prevent overfitting, and a new classification layer (with Softmax). Overall, the data was split with the 80%–20% scheme, as follows: from the 236 total images, 190 were used in training (168 for training; 84 ASY and 84 SY examples, and 22 for internal validation; 11 ASY and 11 SY plaques), and 46 images were used in the final model evaluation (23 ASY and 23 SY cases). In training, we used a minibatch of 12 images and relied on categorical cross entropy (loss function), and the root mean square propagation (“rmsprop”) as optimization algorithm (45) with default settings, starting with an initial learning rate of 0.0001, which we dropped by 0.6 every seven epochs (step decay), tracking the validation loss. We also used data augmentation (rotation range at 20°, horizontal and vertical flips, and shearing in a 50° range) to give more paradigms to the small model, and early stopping (patience at 20 epochs; although seemingly high, experimentation with lower patience levels resulted in premature training).

We repeated the whole process of training of the new set of layers, and testing, three times (three different seeds; 7, 12, and 42), to assess the reproducibility of the results in the model's classification performance. Each time a certain seed was used, it was to prepare three aspects: in training, validation, and testing image generators to prepare the images (data shuffling was also used in generators), in securing reproducibility via setting the *tf.keras.utils.set_random_seed(seed)* [followed by tf.config.experimental.enable_op_determinism()], and in *weight* initialization, via the *tf.keras.initializers.HeUniform(seed)* (preferred due to ReLU) in the dense layer with 128 neurons, and the *tf.keras.initializers.GlorotUniform(seed)* in the final classification layer (due to Softmax).

#### 3.4.3 Model classification performance metrics

We evaluated the model's classification performance measuring the accuracy, the precision per class, the Sensitivity (SE), the Specificity (SP), the F1-score, and the area under the receiver operating characteristic curve (AUC-ROC). We also generated the confusion matrix, showing the percentages of true positives (TP), true negatives (TN), false positives (FP), and false negatives (FN). All metrics used are summarized in (46, 47). The corresponding formulas (1)–(5) of all model performance evaluation metrics, in this study, are shown below:

(1) $$\mathrm{Accuracy}=\frac{TN+TP}{TP+TN+FP+FN}$$

(2) $$\mathrm{Precision}=\frac{TP}{TP+FP}$$

(3) $$\mathrm{Sensitivity}\;(\mathrm{Recall})=\frac{TP}{TP+FN}$$

(4) $$\mathrm{Specificity}\;=\frac{TN}{TN+FP}$$

(5) $$F1\_\mathrm{score}\;=\;\frac{2*\mathrm{Precision}*\mathrm{Recall}}{\mathrm{Precision}+\mathrm{Recall}}$$

### 3.5 Saliency maps and class activation maps

To detect the carotid plaque composition(s) that the model better understands, individually for the ASY and SY class, we explored *smoothed saliency maps*, based on the final FC classification layer, and CAMs, based on the last CONV layer in Xception (frozen). For the first process, we used “SmoothGrad” (40), while in the latter case, we used “f-Score-CAM” (41) (a faster Score-CAM version). We utilized these processes via the visualization toolkit “tf-keras-vis” (48).

#### 3.5.1 Smoothed saliency maps

With “SmoothGrad,” we intended to visualize plaque composition–related areas that influence the model's *final decision*. In (49), we attempted a similar transfer learning-based classification approach, where we used vanilla saliency maps, noticing that the resulting visualizations were noise-rich. SmoothGrad alleviates this problem to some extent, generating multiple saliency maps (varying noise examples of the input), averaging noise, and leading to a smoothed, more interpretable saliency (sensitivity) map. It computes how much difference a small change per pixel in the input image will cause to the final classification score, for the examined class; a computation that needs the derivative (the gradient) of the class activation function. Here, we will visually assess the SmoothGrad maps.

#### 3.5.2 Gradient-free class activation maps

Importantly, we used f-Score-CAM (41), a gradient-free approach, to produce CAMs. It upsamples the CAM of a region in the input image and perturbs the input with this upsampled map, whose importance is calculated by the target score of the masked result. In fact, the upsampled CAM is a mask itself, which receives a normalized smoothing effect ([0,1]) and can be used to perturb the input. Here, we used the “jet” colormap with its continuous color values (50) to give the heatmap (Heatmap; covering the whole GT plaque; GTplq, including all present compositions). We kept the overall heatmap, as *an area of uniform values*, which overlaps with the areas of the plaque and calculated what proportion of each plaque composition is covered by this heatmap and what proportion of each plaque composition area falls inside the heatmap. We maintained the continuous “jet” colormap to *visually* (qualitatively) assess the plaque composition(s) that is important for Xception (its last CONV layer) to understand the ASY or the SY class. Splitting the “jet” map values for a non-linear representation, in six colors (e.g., black, blue, green, yellow, orange, and red), would not serve our current aim. It should be made clear that defining a threshold for the CAM values, to decide on the highly involved plaque composition(s), per class, would probably introduce bias, as it is uncertain what this threshold should be, at the moment.

#### 3.5.3 Detection of compositions driving carotid ultrasound plaque classification

**Figure 2:**
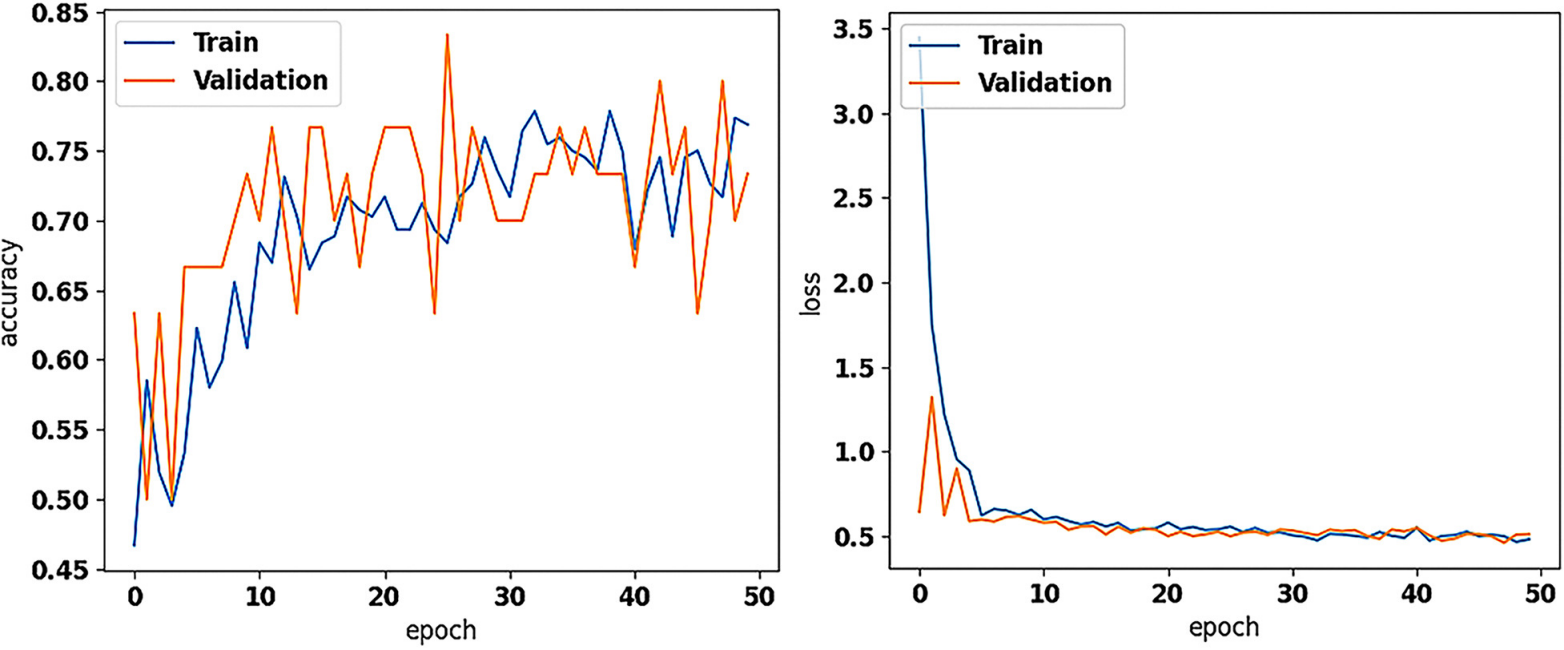
The training and validation accuracy (on the left) and loss values (on the right) of the new model (with the pretrained on ImageNet Xception as base for feature extraction; frozen), trained for asymptomatic vs. symptomatic carotid plaque classification in B-mode ultrasound images (results using 7 and 42 as seeds in training), as performed in this study.

**Figure 3:**
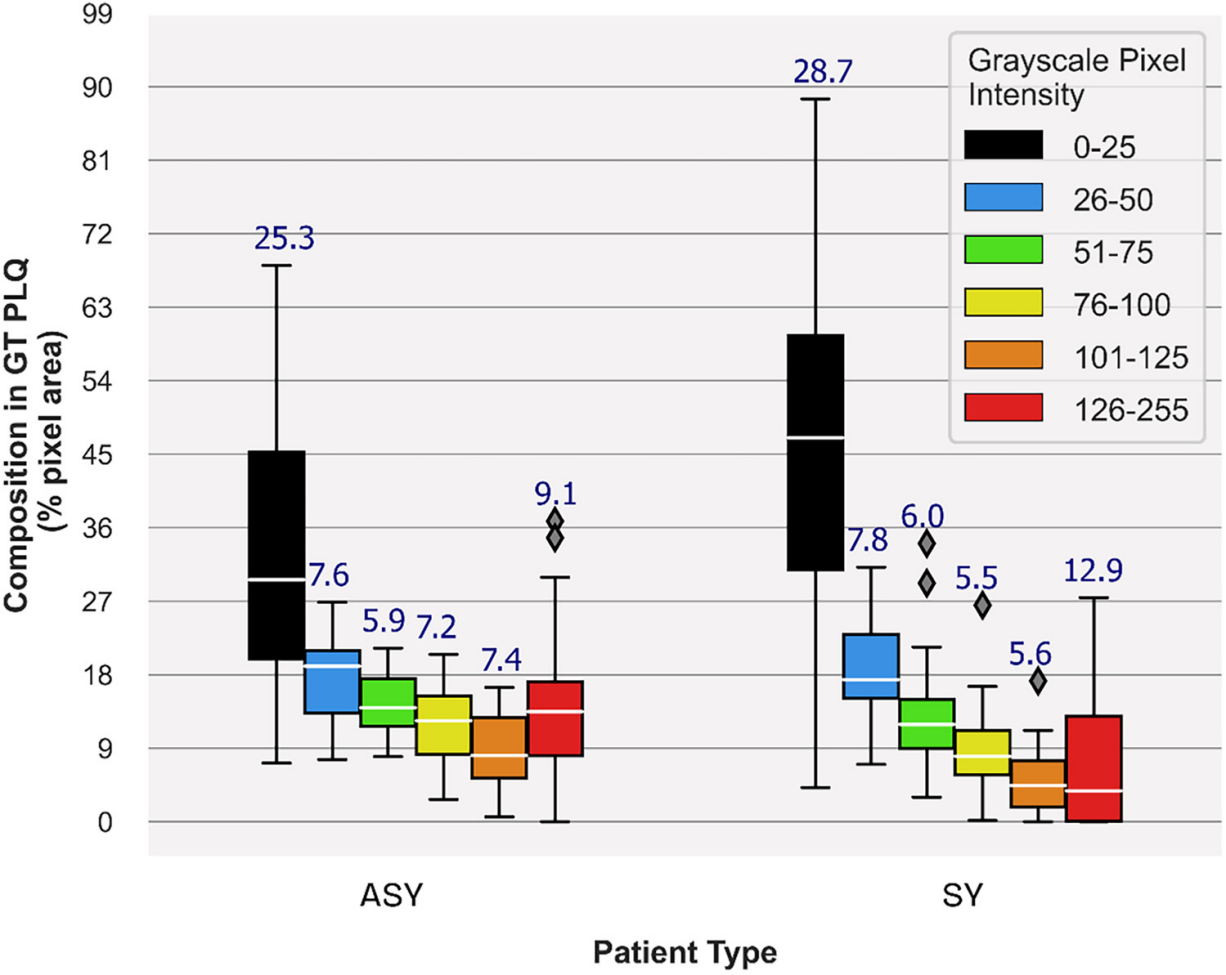
Carotid plaque composition proportions in GTplq total pixel area, depicting plaque synthesis of the 23 ASY and 23 SY evaluated U/S images. Median values are shown in white horizontal lines inside boxes, while above each box, the interquartile range is given. In the legend, each grayscale (GS) value range reflects a specific color contour; lipid cores and JBAs are shown in black (GS **≤** 25), lipid cores with some amount of collagen (histologically it is fibro-fatty tissue) in blue (25 < GS **≤** 50) and green (50 < GS **≤** 75), and calcified areas in orange (100 < GS **≤** 125) and/or red (GS > 125). We depict intermediate areas in yellow (75 < GS **≤** 100), for visualization purposes. ASY, asymptomatic; GT, ground truth; GTplq, ground truth plaque region; JBAs: juxtaluminal black areas; PLQ, plaque; SY, symptomatic; U/S, ultrasound.

For each evaluated GS plaque image, for which the model predicted a class (ASY or SY), we kept the plaque-covering f-Score-CAM heatmap and compared its area with the underlying color-contoured GTplq compositions. We identified the pixel positions of the heatmap and those of each plaque composition (six color contours) and generated groups of measurements to find the influential compositions, in the form of *proportions*. Specifically, the steps we followed were:

1. First, a given plaque image for which a prediction was made is analyzed using f-Score-CAM, which returns a 2D array (512 × 224) Heatmap (the heatmap colors were not compared interchangeably with the GTplq contour colors; there red shows the highest and light blue shows the lowest values, respectively). Our approach was to keep the heatmap colors intact (all colors present) to help understand the areas that are influential in the model's understanding of each class (ASY or SY), visually (qualitatively).
2. Next, only the heatmap area covering the GTplq area is the one used in the main analysis. As this study is of an exploratory nature, we considered all heatmap values, overlapping with the GTplq; although it seems paradoxical, it is partially justified by visualizations in Figures 2, 3 (later), where in the plaque with JBAs (priorly verified by the doctor), the heatmap produces low values, however relevant (see Figure 2, 6th row and 5th column). Importantly, the overall heatmap area was treated as a *uniform map*.
3. Next, we further defined the following measurements, per plaque:
  a. Proportion of GTplq pixel area covered by the heatmap (GTplq-Heatmap).
  b. Proportion of GTplq composition pixel area, with respect to the total GTplq pixel area (BktoGT, BltoGT, GtoGT, YtoGT, OtoGT, and RtoGT, for black, blue, green, yellow, orange, and red, respectively).
  c. Proportion of each heatmap-covered GTplq composition with respect to this GTplq composition's pixel area (HMPinBk, HMPinBl, HMPinG, HMPinY, HMPinO, and HMPinR, respectively).
  d. Proportion of each heatmap-covered GTplq composition pixel area with respect to the overall heatmap's pixel area (HMPBk, HMPBl, HMPG, HMPY, HMPO, and HMPR, respectively).

Measurements (as percentages) in *a*, *b,* and *c* were averaged per composition and across all cases, per class (ASY or SY). We explored the plaque composition area-specific averaged measurement, in *c* (e.g., average HMPinBk, HMPinBl, and the others), across all plaques, as a *descriptive measurement* showing to what extent a composition represents the given class (ASY or SY), from the CNN model's view. Overall, we relied on the averaged measurement, in *d*, to find the *influential* compositions for each class. The different colors in the primary heatmap are used only for visual perception of the overall important plaque compositions, while the six color shades are used to demonstrate the GTplq compositions. A particular focus was drawn on plaques, where the largest present GT composition was also the one mostly covered by the heatmap.

## 4 Results

### 4.1 Transfer learning in carotid ultrasound plaque classification

We trained the new dense layer set at the top of the pretrained Xception (frozen) and evaluated the model's performance with 23 ASY and 23 SY U/S plaque images. Based on Early Stopping, the model's training stopped at 38, 32, and 50 epochs for seeds 7, 12, and 42. The model reached the highest (same) precision and F1-score at 81.8% and 80.0%, and 79.2% and 80.9% for the ASY and SY cases, respectively (Table 3) with seeds 7 and 42. Also, there, the accuracy reached 80.4%, while the SE and SP were found at 82.6% and 78.3%, respectively. The AUC reached 0.80, for all seeds. In Figure 2, we depict the training and validation accuracy and loss, for the model training with seed 42. After all the three model training repetitions (three different seeds), in testing, the mean ± std accuracy, SE, SP, and AUC were found at 80.4 ± 0.0%, 84.1 ± 2.07%, 76.8 ± 2.07% and 0.80 ± 0.0, respectively. The mean ± std precision and F1-Score were found at 82.9 ± 1.5% and 79.7 ± 0.42%, and 78.4 ± 1.08%, and 81.1 ± 0.33% for the ASY and the SY class, respectively. The training and validation accuracy and loss, for the training with seed 42 (best results, as also with seed 7) are shown in Figure 2. We believe that data augmentation possibly caused the primary intense fluctuations between the training and the validation accuracies and losses (see Figure 2, left and right, respectively); the validation loss did not further decrease after a certain value, which might be attributed to the limited number of training images.

**Table 3:** Transfer learning carotid plaque classification metrics (ASY and SY cases), employing the new model (with the pretrained on ImageNet Xception as base; frozen), for three different training sessions, with seed 7, 12, and 42.

Seed	Class	Precision	F1-score	Accuracy	Sensitivity	Specificity	AUC
7	ASY	81.8	80.0	80.4	82.6	78.3	0.80
	SY	79.2	80.9				
12	ASY	85.0	79.1	80.4	87.0	73.9	0.80
	SY	76.9	81.6				
42	ASY	81.8	80.0	80.4	82.6	78.3	0.80
	SY	79.2	80.9				
Mean ± std	ASY	82.9 ± 1.51	79.7 ± 0.42	80.4	84.1 ± 2.07	76.8 ± 2.07	0.80
	SY	78.4 ± 1.08	81.1 ± 0.33				

ASY, asymptomatic; AUC, area under the curve; SY, symptomatic.

### 4.2 Identified compositions contributing to carotid plaque classification

**Figure 4:**
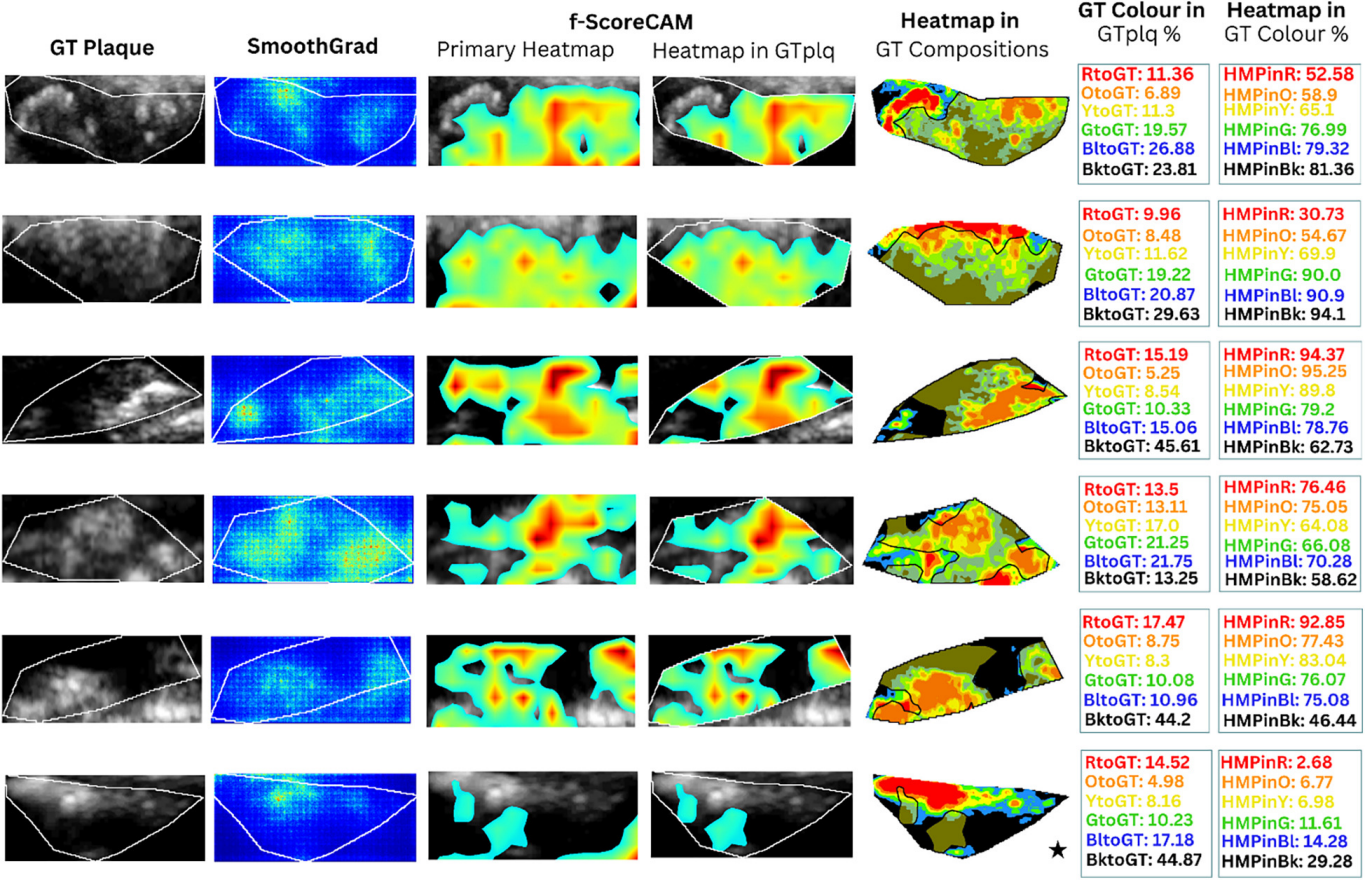
Saliency maps and heatmaps based on faster-Score-CAM, for a sample of six ASY evaluated images, in this study. We show GTplq in standardized, cropped, and resized U/S images (column 1), the SmoothGrad map (column 2), the primary Score-CAM-based heatmap overlaid on the GTplq (column 3), the heatmap within the GTplq (column 4), and the final *uniform* version of the heatmap, overlaid on the individual GTplq compositions, using the six-color contouring method (column 5), according to Kyriacou and Nicolaides (36). Column 6 shows the proportions of GTplq composition pixel areas, with respect to the total GTplq area. Column 7 shows the proportion of each heatmap-covered GTplq composition with respect to this GT composition area. Lipid cores and JBAs are shown in black (GS **≤** 25), lipid cores with some amount of collagen (histologically it is fibro-fatty tissue) in blue (25 < GS **≤** 50) and green (50 < GS **≤** 75), and calcified areas in orange (100 < GS **≤** 125) and red (GS > 125). We depict intermediate areas in yellow (75 < GS **≤** 100), for visualization purposes. *With a star, we signify the existence of a JBA area close to lumen*. ASY, asymptomatic; GT, ground truth; GTplq, ground truth plaque region; HMP, heatmap; JBA, juxtaluminal black area.

**Figure 5:**
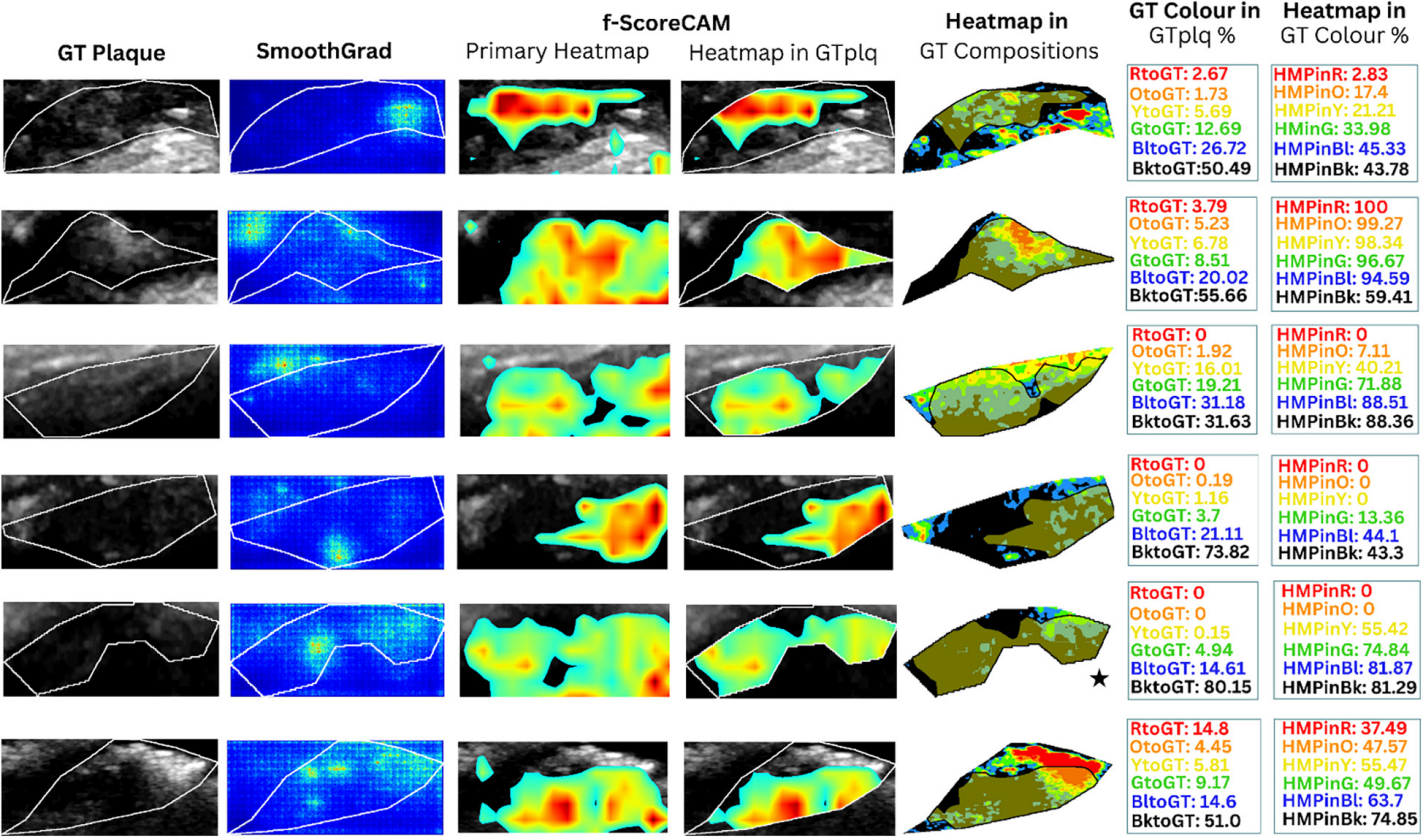
Saliency maps and heatmaps based on faster-Score-CAM, for a sample of 6 SY evaluated images, in this study. We show GTplq in standardized, cropped and resized U/S images (column 1), the SmoothGrad map (column 2), the primary Score-CAM-based heatmap overlaid on the GTplq (column 3), the heatmap within the GTplq (column 4), and the final *uniform* version of the heatmap, overlaid on the individual GTplq compositions, using the six-color contouring method (column 5), according to (36). Column 6 shows the proportions of GTplq composition pixel areas, with respect to the total GTplq area. Column 7 shows the proportion of each heatmap-covered GTplq composition with respect to this GT composition area. Lipid cores and JBAs are shown in black (GS **≤** 25), lipid cores with some amount of collagen (histologically it is fibro-fatty tissue) in blue (25 < GS **≤** 50) and green (50 < GS **≤** 75), and calcified areas in orange (100 < GS **≤** 125) and red (GS > 125). We depict intermediate areas in yellow (75 < GS **≤** 100), for visualization purposes. *With a star, we signify the existence of a JBA area close to lumen*. GT: Ground truth, GTplq: Ground truth plaque region, HMP: Heatmap, JBA: Juxtaluminal Black Area, SY: Symptomatic.

In Figure 3, we provide boxplots showing the overall synthesis of the 23 ASY and the 23 SY evaluated plaques, in this study. We may notice that dark (GS ≤ 25) areas were *largely* present in both the ASY and the SY plaques, compared with the other compositions, although in the SY plaques, dark areas (lipid cores and/or JBAs) were *even larger* than that in the ASY plaques. In Figures 4, 5, we qualitatively present the *saliency maps*, for six ASY and six SY plaques of the evaluated images, showing the plaque areas that contributed to the *final classification score* per class (see Figures 4, 5, column 2; SmoothGrad maps from the new classification layer). We extracted these maps by setting the number of calculating gradient iterations to 20 and the noise level to 0.30 (see “tf-keras-vis” implementation). *Saliency maps*, in many cases, identified key areas per class, different than those extracted by the f-Score-CAM. We noticed a *tendency* of *SmoothGrad* to focus on *calcifications*, in both the ASY and the SY plaques. The saliency maps and visualizations for the rest of the evaluated plaques are in the Supplementary Material (see Figures 1, 2).

In Figures 4, 5 (column 4), we also show the f-Score-CAM-based maps. To use f-Score-CAM (in the last CONV layer in Xception; name: “conv2d_3”), we set the channel number to 3 (upon trials; one can investigate more channels). In Figure 4, based on these maps, we may notice that lipid cores and possibly the JBAs (shown in blue and black, and an asterisk, respectively) were the compositions that largely influenced the understanding of the ASY class. In Figure 5 (SY cases; row 3, 5, and 6), we may see that HMPinBk and HMPinBl were overall higher than those of the other compositions.

In Table 4, we provide the average proportion (%) of the heatmap per plaque composition (HMPBk, HMPBl, HMPG, HMPY, HMPO, and HMPR), with respect to the overall heatmap pixel area. Interestingly, this measurement reveals that dark areas (lipid cores) seem important for the model to understand *both classes*, with the HMPBk in ASY cases found at 30.71 ± 23.75%, and in SY cases at 36.17 ± 30.07%. It seems that dark areas (GS ≤ 25), alone, best describe an SY plaque, as they were largely perceived in the evaluated SY plaques, compared with the calcified areas (GS ≥ 125) (see Table 4, column 3, showing 36.17 ± 30.07% HMPBl vs. 8.35 ± 13.72% HMPO, respectively). Notably, lipid cores, calcified areas, and the collagen and fibrous areas, *together*, contributed to the understanding of the ASY cases, with HMPBl, HMPG, HMPY, HMPO, and HMPR values found at 17.67 ± 9.83%, 13.02 ± 7.12%, 9.86 ± 5.97%, 7.17 ± 5.67%, and 12.84 ± 14.68%, respectively (see Table 4, column 2, bottom). All corresponding average values for these composition measurements in the SY cases were notably *lower* (see Table 4, column 3, bottom right).

**Table 4:** Average proportion of each heatmap-covered gTplq composition area with respect to the f-score-CAM-based (uniform) heatmap total area, from the evaluated U/S images (23 ASY/23 SY).

Measurement	ASY	SY
	mean ± std	mean ± std
% GT Hmap-covered		
GTplq-Heatmap	54.6 ± 25.6	39.0 ± 28.4
Heatmap pixel area corresponding to each GTplq composition (% proportion) mean ± std		
HMPR	**12.84** ± **14.68**	8.35 ± 13.72
HMPO	**7.17** ± **5.67**	5.11 ± 6.12
HMPY	**9.86** ± **5.97**	8.54 ± 9.69
HMPG	**13.02** ± **7.12**	11.81 ± 10.67
HMPBl	***17.67*** ± ***9.83***	*16.96* ± *10.76*
HMPBk	30.71 ± 23.75	**36.17** ± **30.07**

Results in bold show the largest proportion for a given composition, between the two classes (ASY or SY). The underlined results depict the composition with the highest proportion value, within the class. Results in italic show the second more influential composition in each class.

ASY, asymptomatic; HMPBk, HMPBl, HMPG, HMPO, HMPR, and HMPY, proportion of the heatmap-covered black, blue, green, orange, red, and yellow GT pixel area with respect to the Hmap area, respectively; GT, groundtruth; GTplq, groundtruth plaque area; GTplq-Heatmap, proportion of GTplq covered by raw heatmap; SY, symptomatic.

**Figure 6:**
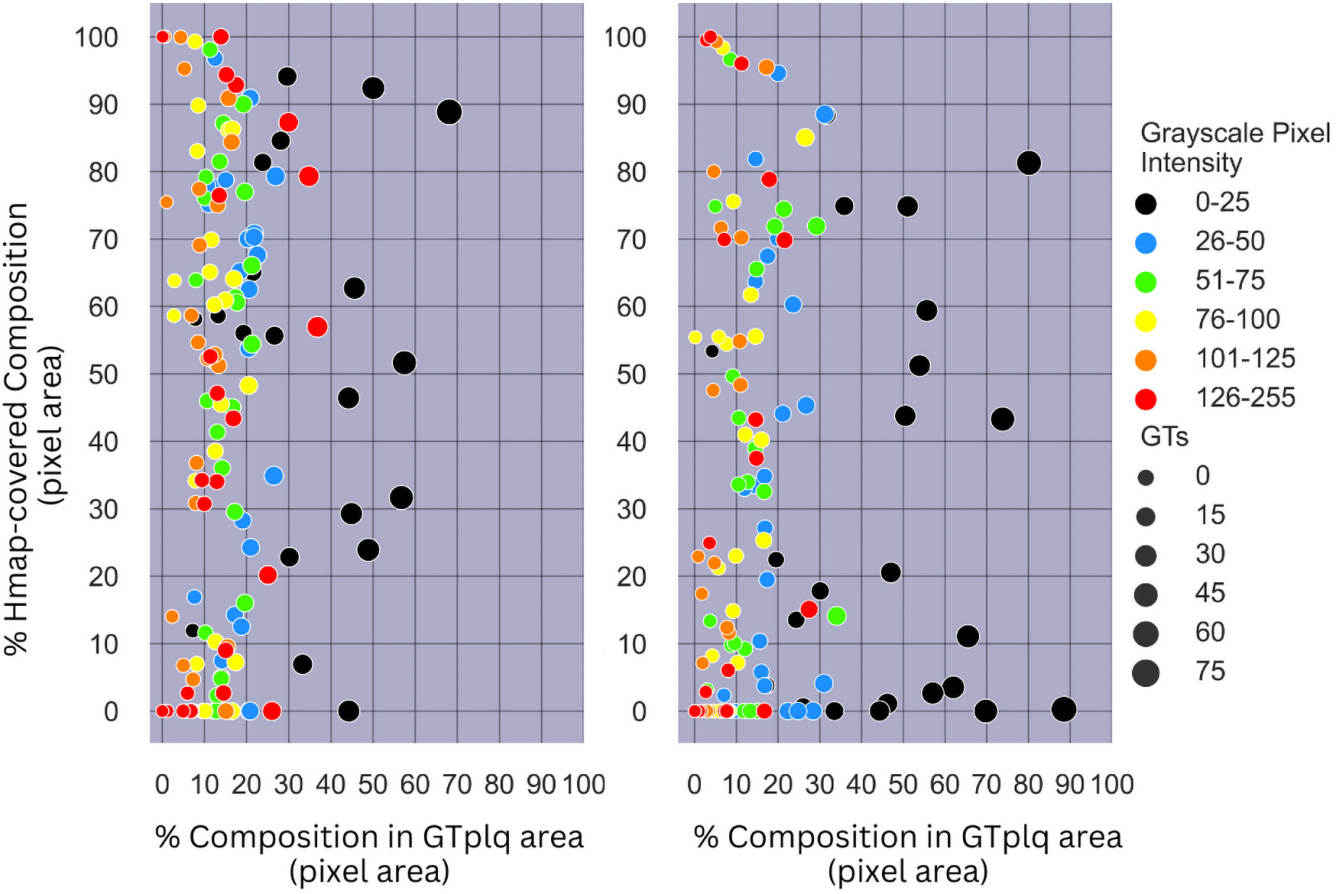
(Left) Scatterplot showing the proportions of all GTplq compositions with respect to the total GTplq pixel area against the proportion of their Hmap-covered area, for all the 23 evaluated ASY plaque images. (Right) Scatterplot showing the proportions of all GTplq compositions with respect to the total GTplq pixel area against the proportion of their heatmap-covered area, for all the 23 evaluated SY plaque images. The larger the circle the larger the proportion a given GTplq composition covers inside its associated plaque. Lipid cores and JBAs are shown in black (GS **≤** 25), lipid cores with some amount of collagen (histologically it is fibro-fatty tissue) are shown in blue (25 < GS **≤** 50) and green (50 < GS **≤** 75), and calcified areas are depicted in orange (100 < GS **≤** 125) and red (GS > 125). We depict intermediate areas in yellow (75 < GS **≤** 100), for visualization purposes. ASY, asymptomatic; GS, grayscale; GT, ground truth; GTplq, ground truth plaque region; SY, symptomatic.

In Figure 6, we provide scatterplots showing the proportions of all GTplq compositions with respect to the total GTplq pixel area, against their proportions with respect to their related heatmaps, for all the ASY (see Figure 6, left) and SY evaluated plaques (see Figure 6, right). There, the larger the circle was the larger the area was that the composition occupied in the given plaque (GTplq). We may notice that *all carotid plaque compositions,* except for the dark areas (GS ≤ 25), generally constituted *smaller areas* in the GTplq, found on multiple levels (HMPColor), for both in the ASY and SY plaques.

For the dark plaque areas (BktoGT; GS ≤ 25) in the ASY plaques, we notice that an heatmap-covered BktoGT was met more often (HMPBk > 20%). For eight SY plaques, there was a 40%<HMPinBk < 85% (see Figure 6, right). The dark areas, although slightly more frequent in the SY plaques, were not consistently found important in all SY cases, whereas when present in the ASY plaques, almost in their entirety, they seemed more *influential*, based on the heatmap coverage (more than half of the ASY BktoGTs had a HMPinBk > 40%).

## 5 Discussion

In this study, we used transfer learning for *feature extraction*, to form a new model for plaque classification in carotid B-mode U/S longitudinal plaques images, followed by the SmoothGrad, and the f-Score-CAM algorithm individually, to discover compositions more explanatory for the ASY or the SY plaques. Despite the limited amount of images, the model yielded a promising classification accuracy and good SE and SP (80.4%, 82.6%, and 78.3%, respectively), with AUC at 0.80. To the best of our knowledge, this is the first study using a well-known pretrained model for feature extraction, for carotid U/S plaque image classification, in *combination* with a process of *quantification* of the area of the underlying plaque compositions, to measure their involvement in the model's understanding of ASY and SY cases, individually, with the help of f-Score-CAM.

In DL-based medical image classification processes, when images (or videos) from different medical centers, observers, or devices are used, image standardization should be an *indispensable preparatory step*; the generalization power of the model does not always guarantee the reliability of the output or that of the statistical analysis results that may follow. The authors strongly believe that reproducible research, comparisons, and results are possible when *image resolution* and *intensity standardization* are priorly performed, in the medical imaging domain. Image resolution standardization (37) alleviates differences in resolution caused by the use of different U/S equipment (devices) and image intensity normalization is highly important to correct brightness or darkness (echodensity levels) of the carotid plaque U/S image. However, not just a carotid U/S image normalization process, but a *clinically relevant* process, where linear scaling is applied with *two reference intensity points*, from the blood area (GS: 0–5) and one from the adventitia area (GS: 198) around the plaque, as proposed in (31), by a highly experienced vascular surgeon. Not all ultrasonographers utilize the same settings (e.g., gain control level) in carotid U/S image capturing.

In (27, 29), a notable model performance was achieved using transfer learning and DL for carotid plaque classification (ASY vs. SY, and high-risk vs. low-risk plaque, respectively), with researchers in (27) including solely the plaque, and researchers in (29) including also an amount of the plaque-surrounding carotid artery area. In both studies, GradCAM was used, although visualizations were only qualitative. In a recent DL-based study (30), large carotid U/S longitudinal image datasets were used with researchers attempting to detect *unstable* plaques, using also GradCAM. There, images came from five different U/S machines; their image intensity normalization method was different from that in (31). We believe that their final results were, to some extent, affected by this choice. Interestingly, their model versions, for the detection and classification of plaques, into *stable* or *unstable*, reached good AUCs in their internal testing, in both tasks, with higher SE and SP in the former DL process (93.2% and 99.2%, and 81.6% and 87.2%, respectively). However, SE was worse on their external dataset, which we believe reflects the *true complexity* of this classification problem. Also, their datasets were imbalanced (especially their training and external testing sets of stable and unstable plaques. From their GradCAM visualizations, we may notice that their model, to some extent, relied on the *hyperechoic areas*, in both the stable and unstable plaques, which contradicts our findings, where the lipid cores (hypoechoic) were more explanatory for the SY cases (and partially for the ASY), based on f-Score-CAM.

In the present study, image standardization might have produced altered model performance per plaque composition, compared with that if we had used the original images. However, if the model was trained on the original images, the classification results might not have been completely trusted, as the plaque compositions' brightness (e.g., JBAs or discrete white areas) might not have been consistent across all inputs. Besides, to consider the JBAs' evaluations, the U/S images should first be *intensity-normalized* (8). Although filtering improves visual assessment by experts and has been found to improve the ASY and SY class separation [based on statistics of texture features (51)], here, we did not consider any filtering method, as this would change the plaque texture features.

In the past, two DL studies (24, 25) delved into GS-based plaque areas, to reveal compositions more effectively learnt by a CNN model, and whether the model could confidently focus on high-risk areas such as JBAs. Although researchers in (24) achieved promising pixel-level SE and SP results, in classification of calcified, fibrous, and lipid-core areas (see Table 1), these metrics were not equally high, reflecting that there might be room for improvement. In (25), researchers discussed in detail a correctly classified ASY plaque, as well as a misclassified true SY plaque, visualizing which compositions were most representative per class. In the latter, they explained that *calcified* areas possibly hindered the classification of SY plaques, as they appeared important for the CNN model's decision, compared with simultaneously present JBAs.

In the present study, SmoothGrad maps revealed that in some SY plaques, the model also highly relied on *calcified* areas. In contrast to (25), we have shown that f-Score-CAM pointed to *wide dark areas* (in some cases, JBAs) in the recognition of SY plaques (see examples in Figure 5, column 5), a composition which, on the other side, in *combination* with the *calcified* and *fibrous-content areas*, seems to help the model understand and correctly classify the ASY cases. Although we have not priorly specified locations of the JBA areas, in this study, we have found that even in ASY plaques, the dark area (GS ≤ 25 or BktoGT) may not only constitute the largest ASY composition area, but it might also be the area that highly influences the identification of the ASY class, in combination with the other areas (see the plaque in asterisk, in Figure 4, row 6, last column; here, we may notice that HMPinBk is not only the highest, at 29.28%, compared with those in the other compositions, but it also corresponds to the largest GTplq composition, with BktoGT at 44.87%). An SY plaque, carrying a JBA, is given in Figure 5 (row 5), with the measurements given in Column 6. There, we may notice that the BktoGT is at 80.15%, from which the HMPinBk is at 81.29%. Many of the 23 evaluated SY plaques, in this study, carried more *homogeneous* and *echolucent* areas, compared with the ASY cases, as also verified in (13).

It is important to mention that GS ≤ 25 areas were not perceived as ASY- or SY-relevant, for every single evaluated plaque in our study, which needs further investigation. To understand the reason behind this:

• It might be beneficial to quantify GS:0 areas inside the GS ≤ 25 areas, as in some cases when on the lumen side, they might be perceived as lumen, practically leading to no CAM values (difficult to predict). A very large dataset should be available to investigate this topic, with manual annotations of JBAs (GS ≤ 25) and GS:0 intra-plaque areas available,
• Measurement of the JBA area (in mm^2^) simultaneously with CAM representations could further enhance the detection of plaques at high risk [see (8)]

Finally, here, an assessment of the contribution of discrete white areas (in the absence of acoustic shadows), in the model's understanding of the ASY or SY plaques, was not possible, as the number of images used for evaluation, as well as the number of plaques having discrete white areas, was limited. Equally, further validation of the current results is needed, with more plaques with JBAs (manually annotated, as GT areas).

In conclusion, in this study we relied on the frozen pretrained Xception, as a feature extractor, to train a new set of two dense layers and classify plaques into ASY or SY, in B-mode U/S longitudinal images, while we also employed f-Score-CAM, to acquire maps to depict plaque compositions or groups of compositions *influential* in the model's understanding of classes (individually). Not surprisingly, the *lipid cores* (depicted as the GS ≤ 25 areas; in some cases, JBAs) seem to hold an *important* role in both the ASY and the SY plaques. The ASY plaques seemed to be recognized, by the model, mostly based on the *coexistence* of *echogenic* and *echolucent* areas. These findings will be further validated, using a larger dataset, and providing increase in confidence scores (based on Score-CAM), while also exploring other explainable approaches for CNNs, focusing on low- vs. high-risk plaque classification.

## 6 Limitations

In the present study, we have identified some limitations. First, the utilized image dataset was not sufficiently large, which we believe had a negative influence on the model's plaque classification performance. We also followed image resizing (advising the resolution-normalized major and minor axis lengths of the plaques), which possibly caused disruptions in contents of some of the smallest plaques. Also, we considered the whole f-Score-CAM-based heatmap, including all values, as setting a threshold in CAM values would possibly introduce some bias, leaving out relevant areas per class. Also, for three SY and two ASY images, the heatmap values did not allow for a visualization; these were the plaques that the model found very difficult to classify. Finally, we could have more JBA- and/or discrete-white-area-rich carotid plaques to train the CNN model.

## Data Availability

The datasets presented in this article are not readily available because the data were collected under strict bioethics approvals and need to be further analyzed thoroughly by our research group, before any public release. Requests to access the datasets should be directed to Efthyvoulos Kyriacou, ehealthlab@cut.ac.cy.
