# Supplementary material for: Transfer learning with class activation maps in compositions driving plaque classification in carotid ultrasound
Source: Front Digit Health. 2025 Jul 9;7:1484231. doi: 10.3389/fdgth.2025.1484231 (PMC12283640; doi:10.3389/fdgth.2025.1484231)
Supplement: Supplementary file 1 [file Datasheet1.pdf]

Supplementary Material

1 Supplementary Figures

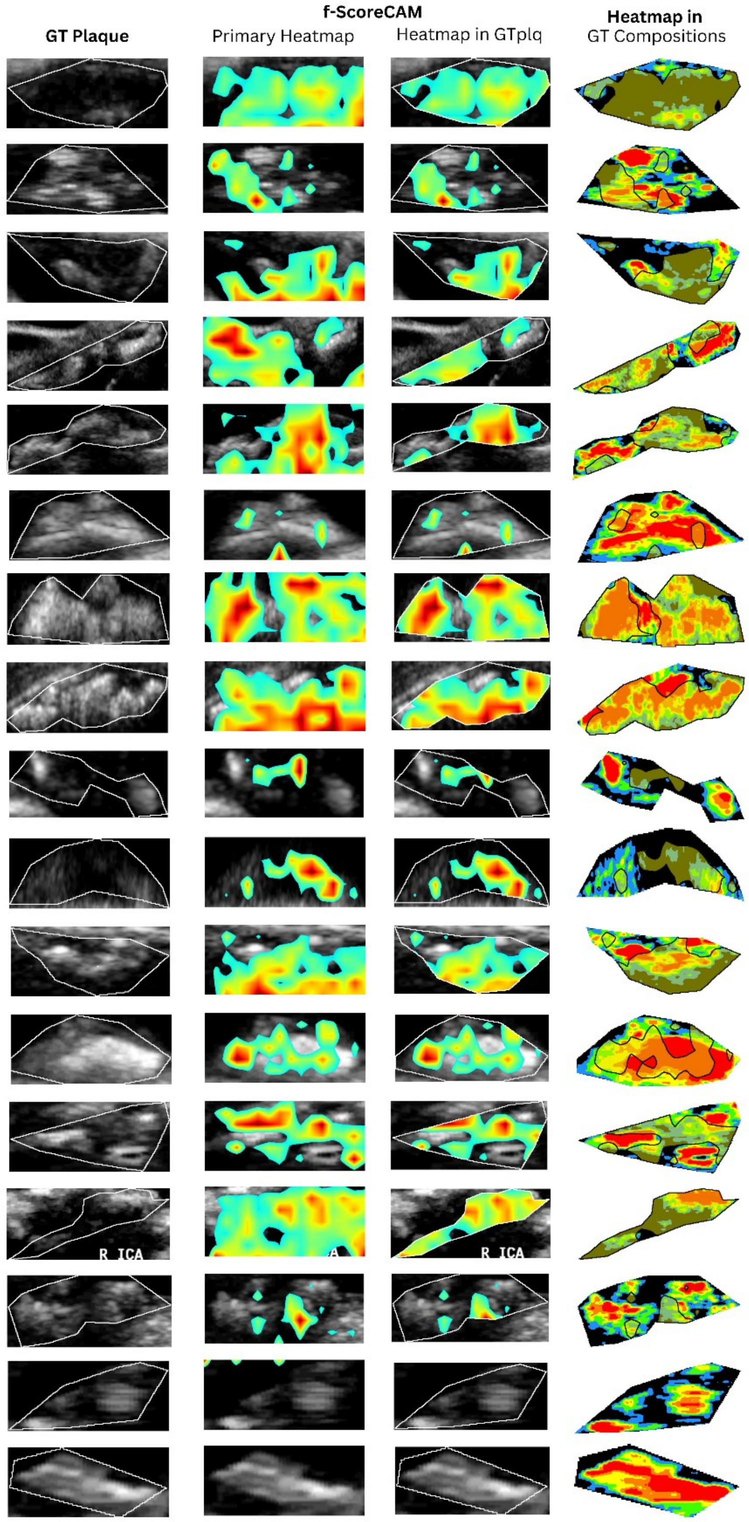

Supplementary Figure 1. Faster-Score-CAM-based Heatmaps, for 17 evaluated Asymptomatic U/S plaque images, in this study. Column 1 shows GTplqs in primary U/S images. Column 2 shows the raw f-Score-CAM Heatmap overlayed on the primary image. Column 3 shows the Heatmap within the GTplq in the primary image and 4 shows the final uniform map, overlayed on individual GTplq compositions, using the 6-colours. Lipid cores and JBAs are shown in black (GS≤25), lipid cores with some amount of collagen (histologically it is fibro-fatty tissue) in blue (25<GS≤50) and green (50<GS≤75), and calcified areas in orange (100<GS≤125) and red (GS>125). We depict intermediate areas in yellow (75<GS≤100), for visualization purposes. GT: Ground truth, GTplq: Ground truth plaque region, Hmap: Heatmap.

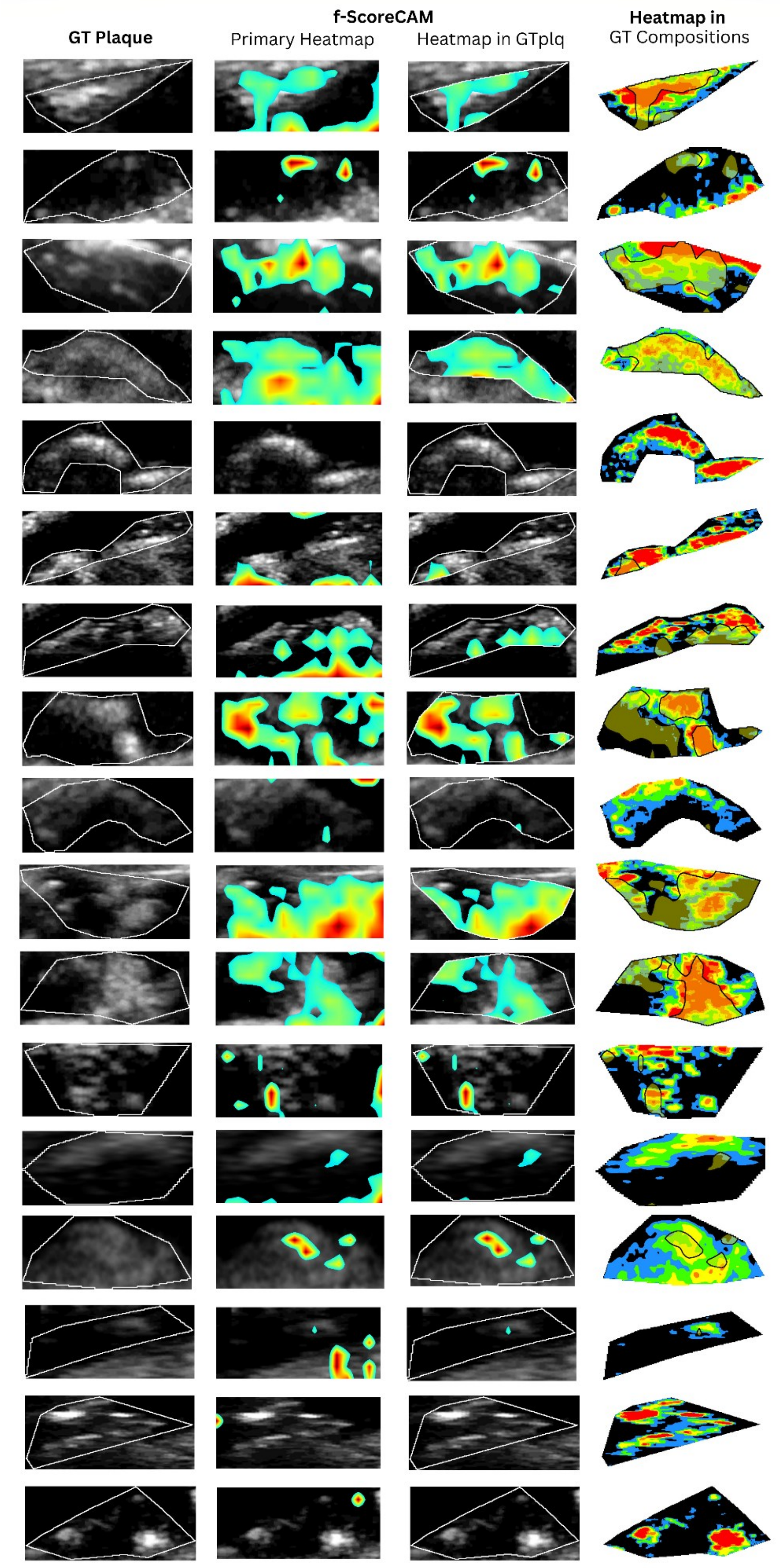

Supplementary Figure 2. Faster-Score-CAM-based Heatmaps, for 17 evaluated Symptomatic U/S plaque images, in this study. Column 1 shows GTplqs in primary U/S images. Column 2 shows the raw f-Score-CAM Heatmap overlayed on the primary image. Column 3 shows the Heatmap within the GTplq in the primary image. Column 4 shows the final uniform version of the Heatmap, overlayed on the individual GTplq compositions, using the 6-colour contouring method. Lipid cores and JBAs are shown in black (GS≤25), lipid cores with some amount of collagen (histologically it is fibro-fatty tissue) in blue (25<GS≤50) and green (50<GS≤75), and calcified areas in orange (100<GS≤125) and red (GS>125). We depict intermediate areas in yellow (75<GS≤100), for visualization purposes. GT: Ground truth, GTplq: Ground truth plaque region, Hmap: Heatmap, JBA: Juxtaluminal Black Area.
